# Expression of Cry1Ab and Cry2Ab by a Polycistronic Transgene with a Self-Cleavage Peptide in Rice

**DOI:** 10.1371/journal.pone.0110006

**Published:** 2014-10-15

**Authors:** Qichao Zhao, Minghong Liu, Miaomiao Tan, Jianhua Gao, Zhicheng Shen

**Affiliations:** 1 State Key Laboratory of Rice Biology, Institute of Insect Sciences, Zhejiang University, Hangzhou, China; 2 College of Life Science, Shanxi Agricultural University, Taigu, China; Instituto de Biotecnología, Universidad Nacional Autónoma de México, Mexico

## Abstract

Insect resistance to *Bacillus thuringiensis* (Bt) crystal protein is a major threat to the long-term use of transgenic Bt crops. Gene stacking is a readily deployable strategy to delay the development of insect resistance while it may also broaden insecticidal spectrum. Here, we report the creation of transgenic rice expressing discrete Cry1Ab and Cry2Ab simultaneously from a single expression cassette using 2A self-cleaving peptides, which are autonomous elements from virus guiding the polycistronic viral gene expression in eukaryotes. The synthetic coding sequences of Cry1Ab and Cry2Ab, linked by the coding sequence of a 2A peptide from either foot and mouth disease virus or porcine teschovirus-1, regardless of order, were all expressed as discrete Cry1Ab and Cry2Ab at high levels in the transgenic rice. Insect bioassays demonstrated that the transgenic plants were highly resistant to lepidopteran pests. This study suggested that 2A peptide can be utilized to express multiple Bt genes at high levels in transgenic crops.

## Introduction

Since first demonstrated in transgenic tobacco in 1987, Bt crystal toxin genes have been widely utilized in transgenic crops for pest management [1], [2]. However, due to the widespread application of Bt toxins, several major insect pests, including fall armyworm (*Spodoptera frugiperda*), dimondback moth (*Plutella xylostella*), maize stalk borer (*Busseola fusca*), cotton bollworm (*Helicoverpa armigera*), western corn rootworm (*Diabrotica virgifera*) and cabbage looper (*Trichoplusia ni*) have developed resistance to Bt toxins in field or greenhouse, which threatens the long-term utilization of Bt crops in the future [3]–[5].

Several strategies have been proposed and/or deployed to cope with the development of insect resistance to Bt toxins, including high-dose/refuge strategy, discovery of novel insecticidal genes with novel modes of actions, modification of used Bt genes and Bt gene stacking [6]–[8]. Bt gene stacking strategy introduces more than one Bt genes into plant and has been demonstrated to be an effective way to delay the development of insect resistance to Bt toxins [9]–[11]. Expression of multiple genes using conventional approaches has several potential limitations, most notably imbalanced expression among different genes and a large T-DNA size required to include multiple genes [12]. Efficient and stoichiometric expression of discrete proteins may be achieved by a polycistronic system involving self-cleaving peptides such as the 2A peptide from foot and mouth disease virus (F2A) or porcine teschovirus-1 (P2A) [12], [13]. When expressed in eukaryotic system, nascent 2A peptide can interact with the ribosome exit tunnel to dictate a stop-codon-independent termination at the final proline codon of 2A peptide [14]. Subsequently translation is reinitiated on the same proline codon. When linked by a 2A peptide coding sequence, different genes are co-expressed from a single open reading frame.

2A self-cleaving peptides have been extensively studied previously [15]. In this study, the DNA encoding F2A or P2A was used to link two potent insecticidal Bt genes, the truncated *Cry1Ab* encoding the N-terminal 648 amino acids of active Cry1Ab endotoxin (Genbank:AAG16877.1) and the full-length *Cry2Ab* encoding Cry2Ab endoxin (Genbank:AAA22342.1) with 634 amino acid residues, to generate a polycistronic gene for co-expression in transgenic rice. Analysis of the obtained transgenic rice lines revealed that discrete Cry1Ab and Cry2Ab were indeed co-expressed at a level approximately comparable to transgenic plants expressing traditional monocistronic Bt genes. Insect bioassays demonstrated that the transgenic rice generated was highly resistant to its target insects.

## Materials and Methods

### Rice cultivar

Elite rice (*Oryza sativa spp. Japonica*) cultivar Xiushui 134 originated from the Jiaxing Academy of Agricultural Science in Zhejiang Province was used for *Agrobacterium*-mediated transformation. The homozygous transgenic rice line KMD1, containing a synthetic truncated *Cry1Ab* gene under control of maize ubiquitin promoter [16], [17], was kindly provided by Dr. Gongyin Ye from Institute of Insect Sciences, ZheJiang University.

### Construction of binary vector for rice transformation

**Figure 1:**
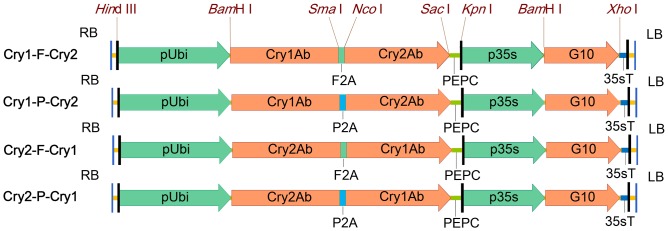
Schematic diagram of T-DNAs for transgenic expression of 2A linked polycistronic genes. Each T-DNA contained two expression cassettes, one for insecticidal polycistronic gene under control of maize ubiquitin-1 promoter (pUbi), the other for glyphosate-tolerant *5-enolpyruvylshikimate-3-phosphate synthase (EPSPS)* gene (G10) under control of Cauliflower mosaic virus 35s promoter (p35s). Cry1Ab and Cry2Ab,synthetic Bt inseciticidal gene; F2A and P2A, synthetic DNAs encoding Foot and Mouth Disease Virus 2A and Porcine teschovirus-1 2A, respectively; RB and LB, right border and left border of T-DNA; PEPC, maize *phosphoenolpyruvate carboxylase* gene terminator; 35sT, Cauliflower mosaic virus 35S gene terminator.

pCambia1300 (Cambia, Canberra, Australia) was used for construction of binary vectors for plant transformation. This vector was first modified by substituting the hygromycin-resistant gene expression cassette with a glyphosate-tolerant *5-enolpyruvylshikimate-3-phosphate synthase* (*EPSPS*) gene expression cassette. The modified vector was named as pCambia1300–GLY, and was further used to clone an expression cassette for a polycistronic gene encoding Cry1Ab and Cry2Ab linked by a 2A peptide (Fig. 1).

Four polycistronic genes of *Cry1Ab* and *Cry2Ab* linked by the coding sequence of F2A or P2A were generated. They were named as *Cry1Ab-F2A-Cry2Ab* (*Cry1-F-Cry2)*, *Cry1Ab-P2A-Cry2Ab* (*Cry1-P-Cry2)*, *Cry2Ab-F2A-Cry1Ab* (*Cry2-F-Cry1)*, and *Cry2Ab-P2A-Cry1Ab* (*Cry2-P-Cry1)*, respectively (Fig. 1). *Cry1-F-Cry2* is a fusion gene, in the order from 5' to 3', of coding sequences of Cry1Ab, F2A, and Cry2Ab (GenBank: KJ716232); *Cry1-P-Cry2* is a fusion gene of Cry1Ab, P2A, and Cry2Ab (GenBank: KJ716233); *Cry2-F-Cry1* is a fusion gene of Cry2Ab, F2A, and Cry1Ab (GenBank: KJ716234); and *Cry2-P-Cry1* is a fusion gene of Cry2Ab, P2A, and Cry1Ab (GenBank: KJ716235).

Maize ubiquitin-1 promoter (pUbi) was obtained by PCR from maize genome with primers pUbi-F (5' attaagcttagcttgcatgcctacagtg 3', with *Hin*dIII restriction site underlined) and pUbi-R (5' taaggatccctctagagtcgacctgca 3', with *Bam*HI restriction site underlined). The pUbi fragment digested with *Hin*dIII and *Bam*HI, the polycistronic gene fragment digested with *Bam*HI and *Kpn*I and the vector 1300-GLY predigested with *Hin*dIII and *Kpn*I were ligated to generate plasmid p1300-Cry1-F-Cry2, p1300-Cry1-P-Cry2,p1300-Cry2-F-Cry1 and p1300-Cry2-P-Cry1, respectively (Fig. 1).

### *Agrobacterium*-mediated transformation

The T-DNA plasmids were separately transformed into *Agrobacterium tumefaciens* LBA4404 by electroporation. The *Agrobacterium*-mediated transformation of rice was carried out according to Hiei Y. *et al*., except that 2 mM glyphosate (Sigma-Aldrich, St. Louis, MO, USA) was used for selection [18].

### RT-PCR analysis of transgenic rice

**Figure 2:**
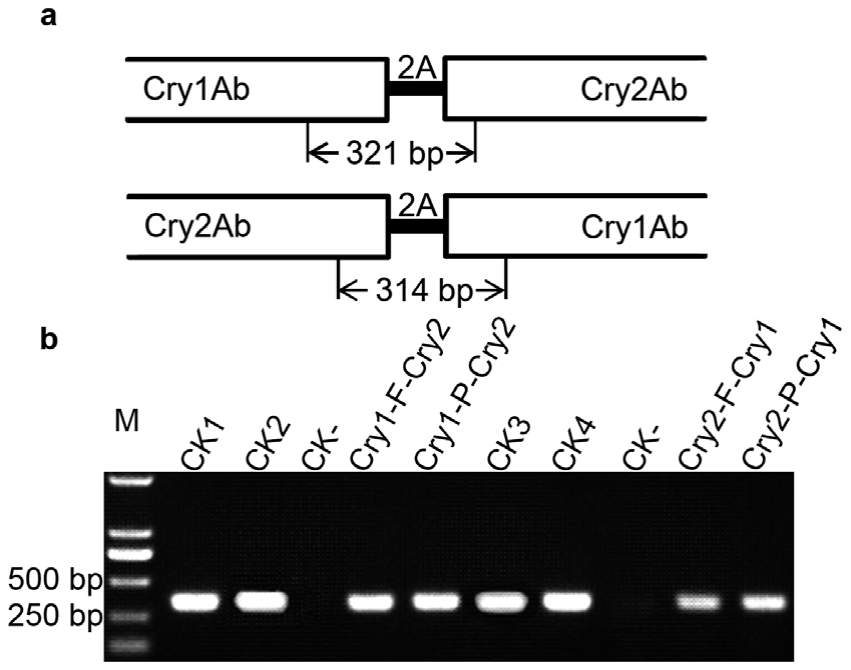
RT-PCR detection of mRNA integrity of the polycistronic genes. **a**, schematic diagram of the fragments amplified by RT-PCR; **b**, electrophoresis analysis of RT-PCR products. CK1, plasmid containing *Cry1-F-Cry2*; CK2, plasmid containing *Cry1-P-Cry2*; CK3, plasmid containing *Cry2-F-Cry1*; CK4, plasmid containing *Cry2-P-Cry1*; CK-, cDNA prepared from non-transgenic rice; M, DNA marker.

Total RNA was extracted from rice leaf with Trizol reagent (Invitrogen, Carlsbad, CA,USA). Concentration of RNA was determined by a spectrophotometer (Thermo Scientific, DE, USA). One µg RNA was treated with Dnase I and used as template to synthesize the first strand of cDNA using cDNA synthesis Kit (Thermo Scientific, DE, USA). Finally the synthesized cDNA was used as PCR template. The primers P1-2-F (5' CAGCGGCAACGAGGTGTACA 3') and P1-2-R (5' TAGGCGTCGCAGATGGTGGT 3') were used to amplify the joint parts in Cry1-F-Cry2 and Cry1-P-Cry2 (Fig. 2**a**). The PCR products were expected to be 321 bp. The primers P2-1-F (5' GCCGCTCGACATCAACGTGA 3') and P2-1-R (5' TCAGGCTGATGTCGATGGGG 3') were used to amplify the joint parts in Cry2-F-Cry1 and Cry2-P-Cry1 (Fig. 2**a**). The PCR products were expected to be 314 bp. The procedure for both PCRs was pre-denaturation at 95°C for 3 min; then 30 cycles of denaturation at 95°C for 40 s, annealing at 50°C for 30 s, and extension at 72°C for 30 s; finally followed with extension at 72°C for 5 min. The PCR products were analyzed by electrophoresis in 1% (w/v) agarose gel.

### Western blot analysis

Leaf sample of 0.01 g collected from 1-month-old rice was frozen in liquid nitrogen for 45 s and disrupted with TissueLyserII (Qiagen, Hilden, Germany) at 40 Hz for 45 s. The sample was then homogenized in 200 µL 1×PBS Buffer by shaking vigorously for 30 s and centrifuged at 12000 rpm for 15 min at 4°C. The supernatants were collected as protein samples for western blot analysis. These samples were separated by electrophoresis on 8% polyacrylamide gel and transferred to PVDF membrane (Pall, Ann Arbor, MI, USA). The blotted membrane was blocked in 5% (w/v) skim milk in Tris Buffer Saline with 0.1% Tween-20 (TBST) for 1 h at room temperature. The membrane was then incubated with primary antibody against either Cry1Ab or Cry2Ab (diluted 1∶1000 in 1% skim milk/TBST) for 1 h at room temperature with gentle shaking. The polyclonal antisera against Cry1Ab and Cry2Ab were prepared from New Zealand white rabbits immunized with purified recombinant Cry1Ab and Cry2Ab from *Escherichia coli*, respectively. After three times of 5 minute wash in TBST, the membrane was incubated with HRP-conjugated secondary antibody (Promega, Madison, WI, USA) diluted at 1∶5000 with 1% skim milk/TBST for 1 h at room temperature with gentle shaking. After another three times of 5 minute wash in TBST, signals were visualized with DAB substrate (Sigma-Aldrich).

### Protein quantification of Cry1Ab and Cry2Ab

Concentrations of Cry1Ab and Cry2Ab in the leaf of transgenic rice at tillering stage were determined by enzyme-linked immunosorbent assay (ELISA) using Envirologix kit AP003 and AP005 (Envirologix, Portland, ME, USA), respectively, according to the manufacturer's recommendation. For each construct, 3 transgenic lines were selected, and the samples prepared with the same method from non-transgenic rice were used to eliminate the basal absorption at 450 nm. After quantification of Cry1Ab and Cry2Ab, the molar ratio of the upstream protein to downstream protein of each transgenic line selected was calculated and analyzed by One-Way ANOVA [19] in SPSS.

### Insect bioassay

Insect bioassays were conducted with cotton bollworm (Bt-susceptible and Cry1Ac-resistant), beet armyworm (*Spodoptera exigua*), striped stem borer (*Chilo suppressalis*) and rice leaf roller (*Cnaphalocrocis medinalis*). For beet armyworm and striped stem borer, detached leaf bioassay was carried out [20]. Two leaf blades from the same line were collected at 2-3 cm length at seedling stage and placed in a 70-mm-diameter petri dish lined with a pre-moistened filter paper. The leaf samples in the petri dish were infested with 10 newly hatched neonates. The petri dishes were then sealed with parafilm membrane and placed in dark at 28°C. The result was recorded after 3 days incubation. For cotton bollworm, detached leaf bioassay was carried out except that one leaf blade was placed in a petri dish and infested with one neonate to avoid cannibalism of cotton bollworm. Each line was infested with a total of 10 neonates. Eggs of Bt-susceptible cotton bollworm (CB-S), beet armyworm and striped stem borer were obtained from Genralpest Biotech (Genralpest Biotech, Bejing, China). Eggs of Cry1Ac-resistant cotton bollworm (CB-RR) were kindly provided by Dr. Kongming Wu from Institute of Plant Protection, Chinese Academy of Agricultural Sciences. For rice leaf roller, whole plant assay was carried out. Tillering-stage rice grown in greenhouse was infested with 10 newly hatched neonates. The result was recorded 2 weeks after infestation. Eggs of rice leaf roller used in the assays were obtained from caged moths collected from rice field. Each assay was repeated for 3 times.

## Results

### Transgene transcribed as a long intact mRNA

About 30 independent transgenic lines were generated via *Agrobacterium*-mediated transformation for each of the four constructs. To investigate whether the transcript of each polycistronic transgene is a long intact mRNA with sequences encoding both Cry1Ab and Cry2Ab, RT-PCR was used to detect the sequences in the area connecting the two Bt genes, which include sequences from both *Cry1Ab* and *Cry2Ab*, and the 2A peptide coding sequence in between (Fig. 2**a**). The RT-PCR products with expected sizes were detected clearly in the transgenic lines from all of the four constructs (Fig. 2**b**), suggesting that each of the polycistronic genes was transcribed into a long intact mRNA as expected.

### Co-expression of discrete Cry1Ab and Cry2Ab from polycistronic transgenes

**Figure 3:**
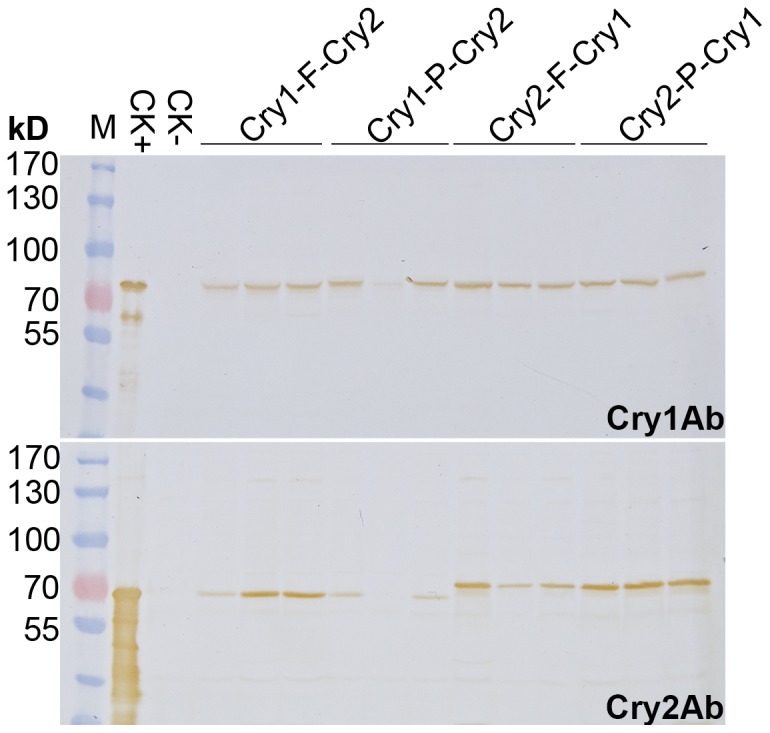
Western bolt analysis of Cry1Ab and Cry2Ab in transgenic rice. Three transgenic lines from each construct were selected for the analysis. Each sample was detected with antiserum against Cry1Ab (upper) and Cry2Ab (bottom) respectively. Cry1Ab or Cry2Ab protein expressed by *E.coli* was used as positive control (CK+). Sample prepared from non-transgenic rice was used as negative control (CK-). M, prestained protein ladder.

Transgenic lines from each construct were analyzed by western blot analysis using antisera against Cry1Ab and Cry2Ab,respectively. When detected with antiserum against Cry1Ab, a band of approximately 72 kD was detected among different transgenic lines, indicating that the Cry1Ab protein was expressed as a discrete protein rather than a fusion protein (Fig. 3 Upper panel). When detected by antiserum against Cry2Ab, a band of about 68∼70 kD was detected among all the transgenic lines (Fig. 3 Lower panel), indicating a discrete Cry2Ab protein was expressed. A possible fusion protein of “Cry-2A-Cry” would be at a size of approximately 147 kD. However, no significant signal of proteins at such size was detected by antiserum against either Cry1Ab or Cry2Ab, suggesting that there was no significant amount of fusion protein expressed in the transgenic rice. The upstream Cry2Ab detected in the Cry2-F-Cry1 and the Cry2-P-Cry1 transgenic lines was slightly larger than the downstream Cry2Ab in the Cry1-F-Cry2 and the Cry1-P-Cry2. This increased size was likely due to the 2A peptide residues attached to the upstream protein during translation,which has been demonstrated in other 2A polycistronic transgenes [21].

### Determination of gene expression level and 2A cleavage efficiency

**Figure 4:**
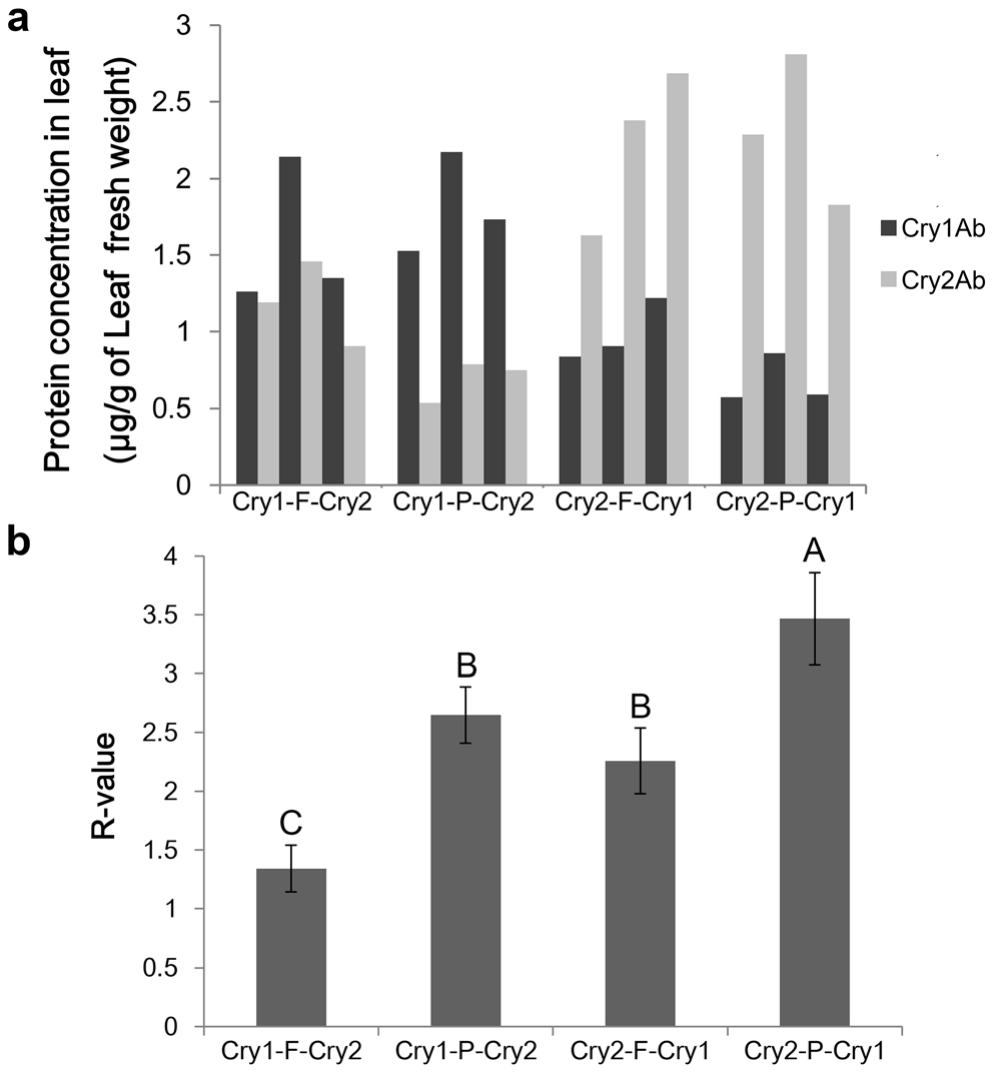
Cry1Ab and Cry2Ab concentrations in different transgenic rice lines and comparison of 2A cleavage efficiency. **a**, concentrations of Cry1Ab and Cry2Ab in leaves of 3 selected transgenic lines from each construct; **b**, R values of different 2A constructs. R value is the molar ratio of the upstream protein to the downstream protein. Different capital letters on each bar indicate extreme significant differences (*p*<0.01, Fisher's least-significant difference and Duncan's multiple range test).

To determine the expression levels of Bt genes with 2A polycistronic transgene and evaluate the cleavage efficiency of 2A peptide in each construct, the amounts of Cry1Ab and Cry2Ab expressed in the transgenic rice leaves were determined by ELISA. Three transgenic lines at tillering stage from each construct were selected. The expression levels of Cry1Ab and Cry2Ab were different among different lines as expected. The concentrations of the soluble Cry1Ab and Cry2Ab in the leaf were in the range of 0.67 to 1.82 µg/g and 0.69 to 2.31 µg/g of leaf fresh weight (LFW), respectively (Fig. 4**a**).

The molar ratio (R) of the upstream protein to the downstream protein of each construct was calculated to estimate the cleavage efficiency. The R value for polycistronic gene using F2A as 2A cleavage peptide was lower than that using P2A (Fig. 4**b**), indicating that the cleavage efficiency of F2A peptide was higher than P2A. The R value of polycistronic gene with Cry1Ab at the upstream was lower than that with Cry2Ab at the upstream (Fig. 4**b**), suggesting that the Cry1Ab at the upstream was likely more efficient in cleavage than the Cry2Ab at the upstream, regardless of whether F2A or P2A was the cleavage peptide in the synthetic construct. The transgenic lines from the construct of Cry1-F-Cry2 had the R value close to 1, indicating that the two Bt proteins were expressed almost equally (Fig. 4**a**).

### Insect-resistant activity of transgenic rice

**Figure 5:**
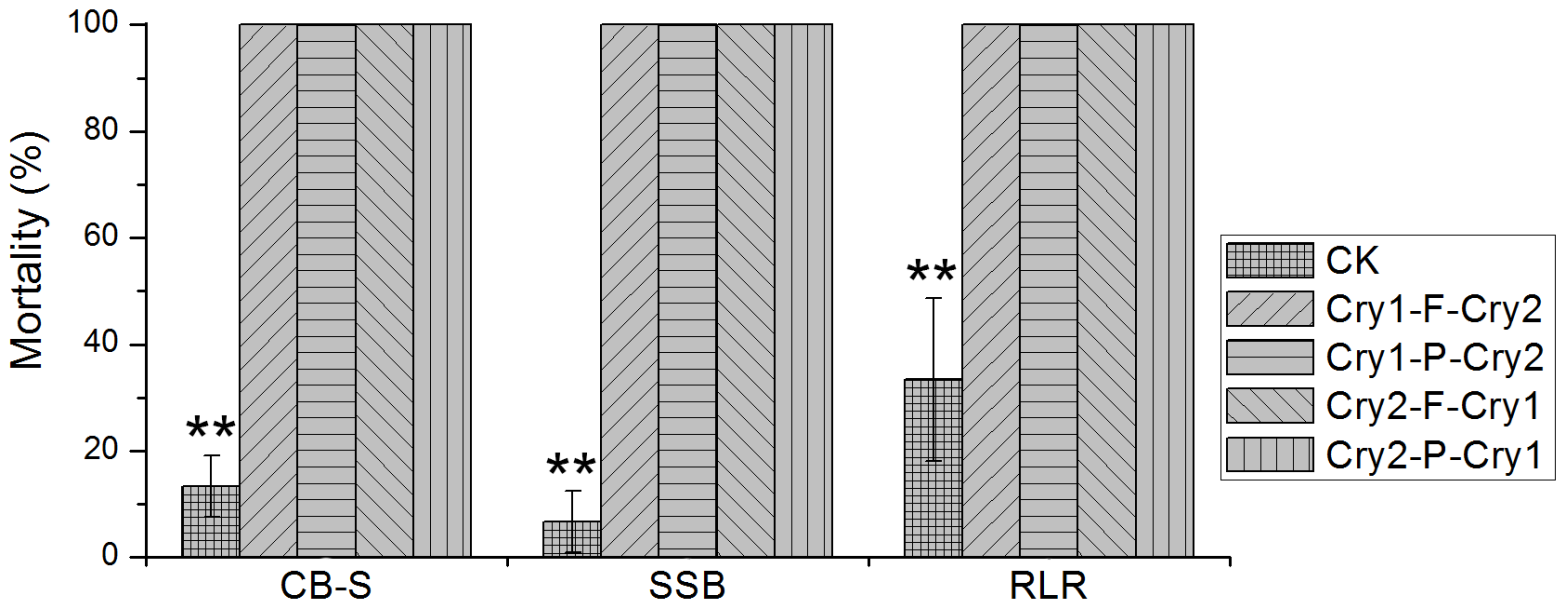
Mortality of Bt-susceptible cotton bollworm, striped stem borer and rice leaf roller feeding on transgenic rice plants. Non-transgenic rice at the same growing stage was used as control (CK). ** on the bar indicate extreme significant differences (*p*<0.01, Kruskal Wallis test). CB-S, Bt-susceptible cotton bollworm; SSB, striped stem borer; RLR, rice leaf roller.

**Figure 6:**
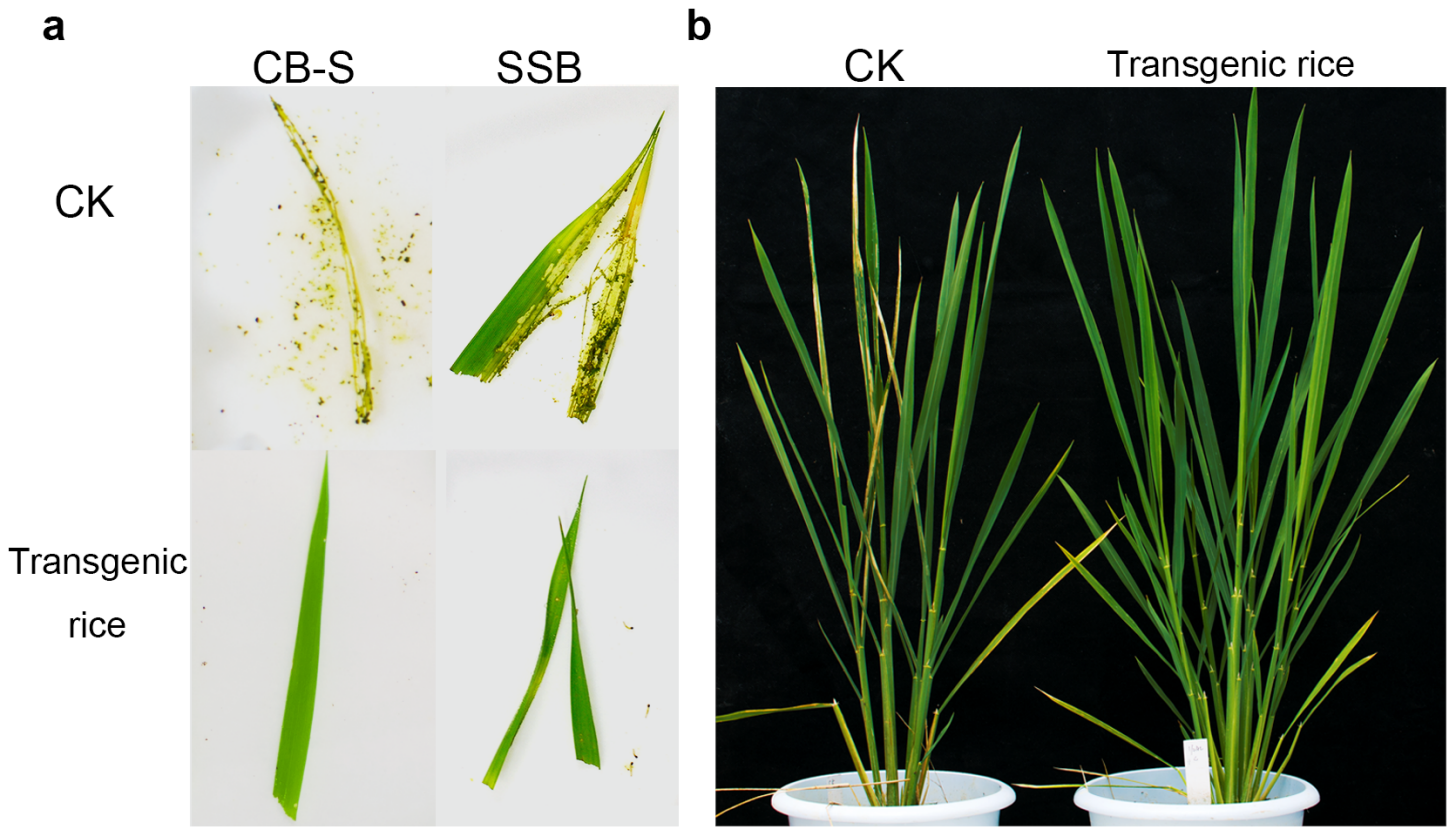
Insect bioassay for transgenic rice with a polycistronic Bt transgene. **a**, bioassay with Bt-susceptible cotton bollworm (CB-S) and striped stem borer (SSB). **b**, bioassay with rice leaf roller. For each assay, age-matched non-transgenic rice was used as control (CK).

The transgenic rice lines were assayed for their insecticidal activity using neonates of CB-S, striped stem borer and rice leaf roller. Three transgenic lines from each construct were selected and age-matched non-transgenic rice plants were used as control. Mortalities of all the 3 insect species feeding on transgenic rice were 100% while those on the non-transgenic rice were much lower (Fig. 5). In the assays with CB-S and striped stem borer, the transgenic leaves were only slightly bitten by the insects while non-transgenic controls were severely consumed (Fig. 6**a**). In the assays with rice leaf roller, no rolled leaf was observed in the transgenic plant while several leaves were rolled in the non-transgenic plant (Fig. 6**b**). The bioassays demonstrated that transgenic rice plants generated from the 2A constructs were highly insect-resistant.

**Figure 7:**
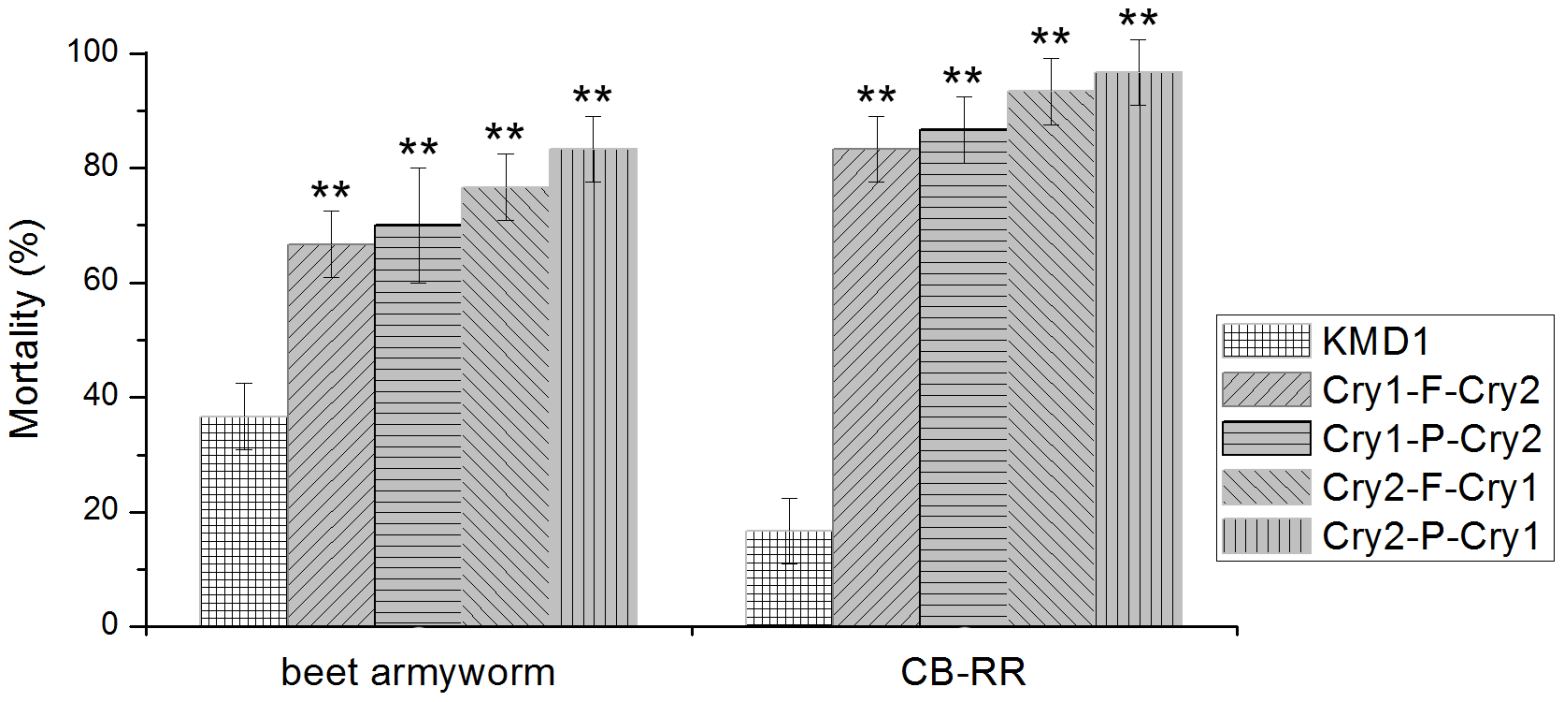
Mortality of beet armyworm and Cry1Ac-resistant contton bollworm feeding on KMD1 and various 2A transgenic rice lines. The transgenic rice KMD1 was used as control. ** on the bar indicate extreme significant difference (*p*<0.01, Fisher's least-significant difference and Duncan's multiple range test). CB-RR, Cry1Ac-resistant cotton bollworm.

The transgenic rice lines were also assayed for their activity against beet armyworm and CB-RR. The KMD1 transgenic rice at the same growing stage was used as control. The mortalities of beet armyworm feeding on the 2A transgenic rice lines were obvious higher than that feeding on KMD1 (*p*<0.01) (Figure 7). The KMD1 showed a rather low activity to CB-RR, while the 2A transgenic rice showed much higher activity (*p*<0.01) (Figure 7).

## Discussion

In this study, Cry1Ab and Cry2Ab, two potent Bt crystal proteins with different receptors in the insect midgut [22], were expressed by the polycistronic transgene using 2A peptide. To our best knowledge, this is the first application of 2A peptide for expressing two Bt genes in a transgenic crop. Utilization of two or more Bt genes simultaneously is desirable for management of insect resistance to Bt toxins as well as for broadening insecticidal spectrum of Bt crops. Self-cleaving 2A peptides and 2A-like sequences have also been found in insect virus, and some of them have high self-cleavage activity [23]. While there is no scientific base for any safety concern for using 2A peptide from mammalian virus, it may be better for public perception to use 2A peptides from insect virus for the development of transgenic rice.

Introduction of multiple genes into plant permits complex and sophisticated manipulation of traits in transgenic crops [2]. The number of genes being introduced into crops for genetic engineering is increasing steadily. For instance, the recently released commercial transgenic corn “Genuity SmartStax” (Monsanto, St Louis, MS, USA) has a total of 8 transgenes for insect-resistance and herbicide-tolerance. With more traits under research and development for transgenic improvement, more genes are expected to be utilized for future transgenic crops. Clearly polycistronic strategy using 2A peptide can simplify the process of gene stacking significantly, as it enables us to introduce multiple genes into plants using a single T-DNA with a single promoter [2]. Moreover, polycistronic strategy alleviates the concern of gene silencing induced by the insertion of homologous promoters or multiple T-DNA sequences into recipient genome [2], [24]. Additionally, equal expressions of different genes could be achieved by the 2A polycistronic strategy [12], [24].

The expression levels of Cry1Ab and Cry2Ab by 2A polycistronic transgene in this study were comparable to traditional monocistronic transgene. The Cry1Ab concentration of transgenic rice KMD1, which is highly resistant to striped stem borer and rice leaf roller [16], was 2.95 µg/g of LFW [25]. The Cry1C concentration in homozygous rice line T1c-19 harboring a synthetic Cry1C gene under control of maize ubiquitin promoter was 1.38 µg/g of LFW at the heading stage [26]. The Cry2A protein concentrations of the homozygous transgenic rice lines harboring a synthetic Cry2 gene driven by maize ubiquitin promoter was reported at the range from 9.65 to 12.11 µg/g of LFW [27]. In the transgenic *indica* rice line harboring both *Cry1Ac* and *Cry2Ab* gene, the Cry2Ab concentration was reported to be around 1 µg/g LFW [28]. Thus, the expression levels of the Cry1Ab and Cry2Ab in the transgenic rice lines obtained by this study were well within the range of the expression levels of the monocistronic transgene reported previously.

Although cotton bollworm was not a pest of rice, it has been long a good target insect for evaluating Bt toxicity. Moreover, cotton bollworm is a good model insect for the study of insect resistance to Bt toxins because of the availabilities of both sensitive and resistant lines to Cry1Ac. The 2A transgenic lines obtained in this study showed high insect-resistant activity against cotton bollworm, either sensitive or resistant to Cry1Ac, while the KMD1 transgenic rice had little activity toward Cry1Ac-resistant cotton bollworm. This suggested that little or no cross resistance existed between Cry1Ac and Cry2Ab and the 2A peptide based transgenic Bt rice will be useful for the management of Bt resistance developed by insect pests.

The 2A transgenic lines obtained in this study conferred significantly higher activity toward beet armyworm than the KMD1 transgenic rice containing Cry1Ab only [16], suggesting that the Cry2Ab expressed in the 2A transgenic lines indeed contributed to the enhancement of insecticidal activity. A similar phenomenon was also observed in transgenic cotton BollgardII. While Bollgard cotton only had moderate toxic activity to beet armyworm, BollgardII cotton showed greatly improved activity due to the addition of Cry2Ab [29].

The upstream proteins expressed with 2A polycistronic strategy are usually attached with the 2A peptide residues at the C-terminus [30]. The western blot analysis in this study appeared to agree with it. However, due to the limitation of the western blot analysis in determination of protein size, C-terminal amino acid sequencing and mass spectrum are required to confirm. While no adverse impact was observed for the utilization of 2A peptide in gene stacking [12], the Bt toxins attached with a 2A peptide will be considered as different proteins according to the regulation of transgenic crops, and thus additional safety studies will be required for commercialization of transgenic crops using 2A cleaving peptide.
